# Labor Income Losses Associated With Heart Disease and Stroke From the 2019 Panel Study of Income Dynamics

**DOI:** 10.1001/jamanetworkopen.2023.2658

**Published:** 2023-03-13

**Authors:** Feijun Luo, Grace Chapel, Zhiqiu Ye, Sandra L. Jackson, Kakoli Roy

**Affiliations:** 1 Division for Heart Disease and Stroke Prevention, National Center for Chronic Disease Prevention and Health Promotion, Centers for Disease Control and Prevention, Atlanta, Georgia; 2 Department of Economics, Tulane University, New Orleans, Louisiana; 3 Center for Healthcare Delivery Science and Innovation, University of Chicago Medicine, Chicago, Illinois; 4 Office of the Director, National Center for Chronic Disease Prevention and Health Promotion, Centers for Disease Control and Prevention, Atlanta, Georgia

## Abstract

**Importance:**

Current estimates of productivity losses associated with heart disease and stroke in the US include income losses from premature mortality but do not include income losses from morbidity.

**Objective:**

To estimate labor income losses associated with morbidity of heart disease and stroke in the US due to missed or lower labor force participation.

**Design, Setting, and Participants:**

This cross-sectional study used 2019 Panel Study of Income Dynamics data to estimate labor income losses associated with heart disease and stroke by comparing labor income between persons with and without heart disease or stroke, after controlling for sociodemographic characteristics and other chronic conditions and considering the situation of zero labor income (eg, withdrawal from the labor market). The study sample included individuals aged 18 to 64 years who were reference persons or spouses or partners. Data analysis was conducted from June 2021 to October 2022.

**Exposure:**

The key exposure was heart disease or stroke.

**Main Outcomes and Measures:**

The main outcome was labor income, measured for the year 2018. Covariates included sociodemographic characteristics and other chronic conditions. Labor income losses associated with heart disease and stroke were estimated using the 2-part model, in which part 1 is to model the probability that labor income is greater than zero and part 2 is to regress positive labor income, with both parts having the same set of explanatory variables.

**Results:**

In the study sample consisting of 12 166 individuals (6721 [52.4%] females) representing a weighted mean income of $48 299 (95% CI, $45 712-$50 885), the prevalence of heart disease was 3.7% and the prevalence of stroke was 1.7%, and there were 1610 Hispanic persons (17.3%), 220 non-Hispanic Asian or Pacific Islander persons (6.0%), 3963 non-Hispanic Black persons (11.0%), and 5688 non-Hispanic White persons (60.2%). The age distribution was largely even, from 21.9% for the age 25 to 34 years group to 25.8% for the age 55 to 64 years group, except for young adults (age 18-24 years), who made up 4.4% of the sample. After adjustment for sociodemographic characteristics and other chronic conditions, persons with heart disease would receive an estimated $13 463 (95% CI, $6993-$19 933) less in annual labor income than those without heart disease (*P* < .001), and persons with stroke would receive an estimated $18 716 (95% CI, $10 356-$27 077) less in annual labor income than those without stroke (*P* < .001). Total labor income losses associated with morbidity were estimated at $203.3 billion for heart disease and $63.6 billion for stroke.

**Conclusions and Relevance:**

These findings suggest that total labor income losses associated with morbidity of heart disease and stroke were far greater than those from premature mortality. Comprehensive estimation of total costs of CVD may assist decision-makers in assessing benefits from averted premature mortality and morbidity and allocating resources to the prevention, management, and control of CVD.

## Key Points

**Question:**

Are labor income losses associated with morbidity of heart disease and stroke in the US?

**Findings:**

In this cross-sectional study of 12 166 individuals aged 18 to 64 years, mean labor income losses were $13 463 for heart disease and $18 716 for stroke in 2018. Total labor income losses were estimated at $203.3 billion for heart disease and $63.6 billion for stroke, far greater than labor income losses associated with premature mortality.

**Meaning:**

These findings suggest that comprehensive estimation of total costs of cardiovascular disease may assist decision-makers in assessing benefits from averted premature mortality and morbidity.

## Teaser

This cross-sectional study estimates labor income losses associated with morbidity of heart disease and stroke among participants in the 2019 Panel Study of Income Dynamics in the US.

## Introduction

Heart disease and stroke, the 2 major categories of cardiovascular disease (CVD), were the first and fifth leading causes of death in the US in 2020, respectively, with 696 962 persons dying of heart disease and 160 264 of stroke.^1^ Approximately 26.1 million people in the US had heart disease or stroke in 2018.^2^ Disparities exist in the burden of heart disease and stroke. There were higher prevalence and poorer management of risk factors^2,3,4^ and higher mortality^5,6^ among non-Hispanic Black persons (compared with non-Hispanic White persons) and socioeconomically disadvantaged persons (compared with affluent persons). Heart disease is associated with physical functional decline.^7^ Stroke, a leading cause of long-term disability,^8^ is associated with complications, such as depression and anxiety, cognitive impairment and dementia, gait instability, and falls and fractures.^2^ These complications may have impacts on employment opportunities, work performance, or career advancement.^9^

Costs of CVD include direct costs of health care and indirect costs of lost productivity.^10^ Lost productivity results from premature mortality, missed workdays, reduced work hours, declined work performance, and missed job opportunities. The American Heart Association reported that annual total costs were $228.7 billion for heart disease and $52.8 billion for stroke in 2017 to 2018, with costs of lost productivity from premature mortality being $119.9 billion for heart disease and $19.4 billion for stroke.^2^ However, those estimates of lost productivity costs did not include income losses from morbidity.

Costs of lost productivity are often quantified through either income loss or costs to employers due to missed workdays and premature mortality among employees. Little is known about lower or lost income in people with CVD, which could result from lower productivity at work or missed job opportunities. Furthermore, previous studies have primarily focused on lost productivity among those who are already employed. For example, a study by Asay et al^11^ quantified the number of missed workdays to estimate the costs of absenteeism associated with hypertension, but the study did not estimate labor income losses among those who could no longer work. A study by Garland et al^12^ calculated the annual earnings losses associated with CVD events from working individuals. Tsao et al^2^ estimated the lost future earnings of individuals who died in 2017 to 2018, but they did not calculate the lost earnings of individuals who are still alive. Few, if any, indirect cost studies have included the labor income loss associated with CVD due to lower labor market participation or lower wages among people with CVD.

This study estimates labor income losses associated with heart disease and stroke by analyzing the 2019 Panel Study of Income Dynamics (PSID) data. This study estimates labor income losses without having to rely on self-reported work absences or estimated unproductive worktime. Furthermore, after controlling for sociodemographic characteristics and other chronic conditions and considering the situation of zero labor income (eg, withdrawal from the labor market), this study provides a direct estimate of labor income losses associated with heart disease and stroke.

## Methods

This cross-sectional study was deemed exempt from review and informed consent by the Centers for Disease Control and Prevention institutional review board because it was an observational study of deidentified participants from publicly available data. We followed the Strengthening the Reporting of Observational Studies in Epidemiology (STROBE) reporting guideline for cross-sectional studies.

### Data Source

We used the family file and the individual file of 2019 PSID for this study. The PSID is the longest running longitudinal survey of US households. It was originally designed to study the dynamics of income and poverty and started in 1968 with a nationally representative sample of more than 18 000 individuals from 5000 families in the US.^13^ Over 50 years, more than 82 000 individuals have participated in the PSID. The PSID has provided cross-sectional individual weights since 1997 and calibrated those weights to the population characteristics from the Current Population Survey or American Community Survey for the respective year.^14^ Beginning in 1999, the PSID switched from annual to biennial interviewing and started collecting information on chronic diseases.

The 2019 PSID includes 9569 families and 26 084 individuals.^15^ The family file contains most variables, including all family-level information and detailed information on the reference person and the spouse or partner.^15^ The individual file includes information on every individual in an interviewed family, but the information is relatively limited. Because PSID reported chronic conditions only for reference persons and their spouses or partners, we focused the study sample on reference persons and their spouses or partners and excluded other family members. We merged labor income, chronic conditions, and sociodemographic characteristics of reference persons and their spouses or partners from the family file with the corresponding persons’ complex survey design variables (stratum, cluster, and weight) from the individual file. Because many people are no longer in the labor force after age 65 years, we restricted the study sample to persons aged 18 to 64 years.

### Measures of Labor Income

Labor income, collected in the 2019 PSID covering the preceding tax year (2018), was the sum of the following components: wages and salaries, bonus income, overtime income, tips, commission income, professional practice income, additional job income, and miscellaneous labor income.^16^ PSID imputed a component of labor income when it had a value of do not know, refused, or outside the range of plausible values.^15,17^ Wages and salaries were the most important component of labor income (98% share), and the 2019 PSID imputed wages and salaries for approximately 5% of reference persons and 2% of spouses or partners.^15^

### Measures of Heart Disease and Stroke

Heart disease was determined by 2 questions: heart attack (“Has a doctor or other health professional EVER told you that you had a heart attack?”) and coronary heart disease, angina, or congestive heart failure (“Has a doctor or other health professional EVER told you that you had coronary heart disease, angina, congestive heart failure?”).^16^ We coded a person as having heart disease if that person answered yes to either question. Stroke was determined by answering yes to the question: “Has a doctor or other health professional EVER told you that you had a stroke?”^16^

### Measures of Sociodemographic Characteristics and Other Chronic Conditions

We included the following sociodemographic characteristics: age group (18-24, 25-34, 35-44, 45-54, and 55-64 years), sex (male and female), race and ethnicity (Hispanic, non-Hispanic Asian or Pacific Islander, non-Hispanic Black, non-Hispanic White, and others [including non-Hispanic American Indian or Alaska Native persons, individuals with multiple races or other race, and unknown]), region (Northeast, North Center, South, West, and outside US), marital status (currently married or never married, widowed, divorced, or separated), education (less than college, at least some college education, and missing), and health insurance (currently have, currently do not have, and missing). We created the specific race and ethnicity categories by combining participants’ responses to survey questions on race and ethnicity. Race and ethnicity were assessed because they are associated with labor income.

To account for comorbidities of heart disease and stroke, we entered the number of other chronic conditions (0, 1, 2, ≥3, and missing) as a covariate. Other chronic conditions included arthritis, asthma, cancer, diabetes, lung disease, and psychiatric conditions.

### Statistical Analysis

For descriptive analyses, we calculated the prevalence of heart disease and stroke among persons aged 18 to 64 years and frequencies of sociodemographic characteristics and other chronic conditions. We reported mean labor income for all persons aged 18 to 64 years and for subgroups by sociodemographic and disease characteristics. We compared percentages of zero labor income between persons with and without heart disease or stroke.

To estimate labor income losses associated with heart disease and stroke, we used the 2-part model. The 2-part model is often used in modeling health care cost as a dependent variable.^18^ Like health care cost, labor income also has excess zeros, right-skewness, and nonnegativity. In our 2-part model, part 1 is to model the probability that labor income is greater than zero and part 2 is to regress positive labor income, with both parts having the same set of explanatory variables. The explanatory variables include the key exposure of interest (heart disease or stroke), sociodemographic characteristics, and other chronic conditions. We used *twopm*, a Stata software (StataCorp) command developed by Belotti et al,^19^ to conduct the analysis. We specified a probit model for the first part and a generalized linear model with the log link and γ distribution for the second part. After estimation, we used *margin* commands to produce average marginal effects (AMEs) of heart disease and stroke on labor income and then marginal effects (MEs) by age group, sex, and race and ethnicity.

We considered models with and without the interaction terms between heart disease and stroke and age group or sex or race and ethnicity. We used one type of models as the main models and the other type of models in a sensitive analysis. We excluded diabetes as a comorbidity in another sensitivity analysis because diabetes is a risk factor associated with heart disease and stroke.

We performed all descriptive analyses using survey procedures in SAS software version 9.4 (SAS Institute) to incorporate the complex survey design (stratum, cluster, and weight) of PSID. We conducted all modeling and postestimation analyses using survey commands in Stata version 15. A 2-tailed *P* < .05 was considered statistically significant.

## Results

### Characteristics of Study Participants

The study sample consisted of 12 166 individuals (6721 [52.4%] females) aged 18 to 64 years who were reference persons or spouses or partners in the 2019 PSID. The prevalence of heart disease was 3.7%, and the prevalence of stroke was 1.7% (Table 1). The age distribution was largely even, from 21.9% for the age 25 to 34 years group to 25.8% for the age 55 to 64 years group, except for young adults (age 18-24 years), who made up 4.4% of the sample. There were 1610 Hispanic persons (17.3%), 220 non-Hispanic Asian or Pacific Islander persons (6.0%), 3963 non-Hispanic Black persons (11.0%), and 5688 (60.2%) non-Hispanic White persons. Close to 60% of persons (7271 persons [57.8%]) had at least some college education and 4746 persons (41.3%) had no college education. Most persons (6841 persons [60.9%]) were married, and 5324 persons (39.1%) were never married, widowed, divorced, or separated. Additionally, most persons (11 154 persons [92.7%]) had health insurance. Although 7953 persons (62.5%) reported no other chronic conditions, more than 10% of persons reported multiple conditions: 937 persons (8.7%) had 2 other chronic conditions and 404 persons (3.9%) had 3 or more (Table 1).

**Table 1.** Characteristics of the 2019 Panel Study of Income Dynamics Participants Aged 18 to 64 Years

Characteristic^a^	Unweighted, No.	Weighted, % (95% CI)
Heart disease condition^b^		
Yes	376	3.7 (3.1-4.2)
No	11 735	96.3 (95.8-96.9)
Stroke condition^c^		
Yes	207	1.7 (1.3-2.1)
No	11 904	98.3 (97.9-98.7)
Age, y		
18-24	675	4.4 (4.0-4.8)
25-34	3471	21.9 (20.5-23.4)
35-44	3491	24.3 (22.9-25.7)
45-54	2165	23.6 (22.2-25.1)
55-64	2364	25.8 (24.5-27.1)
Sex		
Male	5445	47.6 (46.7-48.6)
Female	6721	52.4 (51.4-53.3)
Race and ethnicity		
Hispanic	1610	17.3 (14.9-19.7)
Non-Hispanic		
Asian/Pacific Islander	220	6.0 (4.3-7.8)
Black	3963	11.0 (8.7-13.2)
White	5688	60.2 (56.3-64.0)
Other^d^	685	5.5 (4.8-6.3)
Region		
Northeast	1458	17.1 (13.8-20.4)
North Central	2989	21.2 (19.1-23.2)
South	5424	37.4 (34.5-40.3)
West	2231	23.7 (20.3-27.0)
Outside US	64	0.7 (0.4-0.9)
Education		
<College	4746	41.3 (39.0-43.6)
≥Some college education	7271	57.8 (55.5-60.2)
Missing	149	0.9 (0.7-1.1)
Marital status^e^		
Married	6841	60.9 (59.1-62.6)
Never married, widowed, divorced, or separated	5324	39.1 (37.4-40.9)
Health insurance		
Currently have	11 154	92.7 (91.9-93.5)
Currently do not have	940	6.8 (5.9-7.6)
Missing	72	0.5 (0.3-0.7)
Other chronic conditions, No.		
0	7953	62.5 (60.9-64.1)
1	2804	24.3 (23.1-25.6)
2	937	8.7 (7.9-9.4)
≥3	404	3.9 (3.2-4.6)
Missing	68	0.6 (0.3-0.8)

^a^ Data were extracted from the 2019 Panel Study of Income Dynamics. The study participants included 12 166 individuals aged 18 to 64 years who were reference persons or spouses or partners.

^b^ Heart disease condition was missing for 55 participants.

^c^ Stroke condition was missing for 55 participants.

^d^ Includes non-Hispanic American Indian or Alaska Native persons, individuals with multiple races or other race, and unknown.

^e^ Marital status was missing for 1 participant.

### Labor Income in Total and by Characteristics

Among 12 166 individuals, the weighted mean labor income was $48 299 (95% CI, $45 712-$50 885) (Table 2). Labor income differed substantially by cardiovascular disease status, with a mean of $27 285 (95% CI, $21 371-$33 199) for persons with heart disease vs $49 129 (95% CI, $46 546-$51 712) for persons without heart disease (*P* < .001) and $17 880 (95% CI, $10 050-$25 710) for persons with stroke vs $48 869 (95% CI, $46 286-$51 452) for persons without stroke (*P* < .001). Mean labor income also differed substantially by the number of chronic conditions; for example, persons with no other chronic conditions had a mean income of $53 669 (95% CI, $50 580-$56 758), compared with $19 595 (95% CI, $13 156-$26 035) for persons with 3 or more other chronic conditions. Also noteworthy were the mean labor income comparisons between males and females, with males earning a mean of $63 772 (95% CI, $59 639-$67 905) and females earning a mean of $34 215 (95% CI, $32 704-$35 727), and between persons with education below college and with at least some college education ($28 864 [95% CI, $26 995-$30 732] vs $62 241 [95% CI, $58 779-$65 703]) (Table 2).

**Table 2.** Labor Income of 2019 Panel Study of Income Dynamics Participants Aged 18 to 64 Years in Total and by Subgroup

Labor income	Unweighted, No.	Weighted, mean (95% CI), $
Total	12 166	48 299 (45 712-50 885)
Heart disease condition^a^		
Yes	376	27 285 (21 371-33 199)
No	11 735	49 129 (46 546-51 712)
Stroke condition^b^		
Yes	207	17 880 (10 050-25 710)
No	11 904	48 869 (46 286-51 452)
Age, y		
18-24	675	25 242 (22 618-27 867)
25-34	3471	43 115 (40 613-45 617)
35-44	3491	55 740 (51 724-59 756)
45-54	2165	56 085 (51 563-60 607)
55-64	2364	42 487 (38 043-49 932)
Sex		
Male	5445	63 772 (59 639-67 905)
Female	6721	34 215 (32 704-35 727)
Race and ethnicity		
Hispanic	1610	33 163 (30 595-35 732)
Non-Hispanic		
Asian/Pacific Islander	220	71 860 (55 861-87 859)
Black	3963	34 535 (31 928-37 141)
White	5688	53 481 (50 404-56 557)
Others^c^	685	40 857 (35 146-46 568)
Region		
Northeast	1458	56 951 (49 281-64 621)
North Central	2989	45 269 (39 828-50 709)
South	5424	45 878 (42 552-49 204)
West	2231	48 629 (43 901-53 357)
Outside US	64	46 678 (8999-84 356)
Education		
<College	4746	28 864 (26 995-30 732)
≥Some college education	7271	62 241 (58 779-65 703)
Missing	149	43 237 (32 945-53 529)
Marital status^d^		
Married	6841	55 043 (51 870-58 217)
Never married, widowed, divorced, or separated	5324	37 820 (35 004-40 635)
Health insurance		
Currently have	11 154	50 090 (47 405-52 774)
Currently don’t have	940	24 406 (22 297-26 515)
Missing	72	40 509 (10 038-70 980)
No. of other chronic conditions		
0	7953	53 669 (50 580-56 758)
1	2804	45 779 (41 937-49 621)
2	937	29 992 (25 918-34 066)
≥3	404	19 595 (13 156-26 035)
Missing	68	42 547 (19 012-66 083)

^a^ Heart disease condition was missing for 55 participants.

^b^ Stroke condition was missing for 55 participants.

^c^ Includes non-Hispanic American Indian or Alaska Native persons, individuals with multiple races or other race, and unknown.

^d^ Marital status was missing for 1 participant.

### Percentages of Zero Labor Income

Among 376 persons with heart disease, 170 reported zero labor income (Table 3). The weighted percentage of zero labor income among persons with heart disease was 46.9% (95% CI, 38.4%-55.4%), more than 2.5 times that among persons without heart disease, 18.2% (95% CI, 16.8%-19.5%). The weighted percentage of zero labor income among persons with stroke was even higher, 58.9% (95% CI, 48.8%-69.0%), more than 3 times that among persons without stroke, 18.5% (95% CI, 17.1%-19.9%).

**Table 3.** Percentages of 2019 Panel Study of Income Dynamics Participants Aged 18 to 64 Years Reporting Zero Labor Income

Group	Total	Reporting zero labor income	
		Unweighted, No.	Weighted, % (95% CI)
People with heart disease	376	170	46.9 (38.4-55.4)
People without heart disease	11 735	1849	18.2 (16.8-19.5)
People with stroke	207	116	58.9 (48.8-69.0)
People without stroke	11 904	1902	18.5 (17.1-19.9)

### Labor Income Losses Estimated From 2-Part Models

The models without interaction terms between heart disease or stroke and age group or sex or race and ethnicity were selected as the main models because most interaction terms were not statistically significant. The results were based on models without interaction terms. The parameter estimates are reported in eTable 1 and eTable 2 in Supplement 1.

#### Mean Labor Income Losses

Table 4 reports estimates of mean labor income losses associated with heart disease and stroke from 2-part models. Model 1 found that the AME of heart disease was −$13 463 (95% CI, −$19 933 to −$6993; *P* < .001), which means persons with heart disease would earn a mean of $13 463 less on labor income than those without heart disease, after sociodemographic characteristics and other chronic conditions were controlled for. To put it into context, $13 463 amounts to more than 25% of the model-estimated mean labor income, $47 942. There was an even more substantial income shortfall for persons with stroke. Model 2 found that the AME of stroke was −$18 716 (95% CI, −$27 077 to −$10 356; *P* < .001), which means persons with stroke would earn a mean of $18 716 less on labor income than those without stroke, almost 40% of the model-estimated mean labor income of $47 941.

**Table 4.** Mean Labor Income Losses Associated With Heart Disease and Stroke^a^

Characteristic	Model 1, heart disease (n = 12 110)		Model 2, stroke (n = 12 110)	
	Estimate (95% CI), $	*P* value	Estimate (95% CI), $	*P* value
Heart disease	−13 463 (−19 933 to −6993)	<.001	NA	NA
Stroke	NA	NA	−18 716 (−27 077 to −10 356)	<.001
Age group, y				
18-24	0 [Reference]	NA	0 [Reference]	NA
25-34	12 278 (8677-15 879)	<.001	12 331 (8724-15 938)	<.001
35-44	22 167 (17 690-26 645)	<.001	22 119 (17 631-26 606)	<.001
45-54	25 595 (20 840-30 350)	<.001	25 507 (20 756-30 257)	<.001
55-64	14 960 (9739-20 181)	<.001	14 709 (9489-19 930)	<.001
Sex				
Male	0 [Reference]	NA	0 [Reference]	NA
Female	−28 637 (−31 249 to −26 025)	<.001	−28 310 (−30 876 to −25 743)	<.001
Race/ethnicity				
Hispanic	−13 595 (−16 589 to −10 601)	<.001	−13 571 (−16 603 to −10 539)	<.001
Non-Hispanic				
Asian or Pacific Islander	6654 (−3960 to 17 269)	.22	6863 (−3743 to 17 468)	.20
Black	−11 553 (−15 110 to −7997)	<.001	−11 427 (−15 006 to −7848)	<.001
White	0 [Reference]	NA	0 [Reference]	NA
Other^b^	−8418 (−12 190 to −4645)	<.001	−8104 (−11 794 to −4415)	<.001
Region				
Northeast	0 [Reference]	NA	0 [Reference]	NA
North Central	−8847 (−14 350 to −3343)	.002	−8786 (−14 250 to −3322)	.002
South	−7666 (−11 926 to −3406)	.001	−7562 (−11 759 to −3364)	.001
West	−7601 (−12 288 to −2915)	.002	−7430 (−11 990 to −2870)	.002
Outside US	−22 488 (−42 799 to −2178)	.03	−22 313 (−42 723 to −1904)	.03
Education				
<College	−28 430 (−31 567 to −25 292)	<.001	−28 436 (−31 576 to −25 295)	<.001
≥Some college education	0 [Reference]	NA	0 [Reference]	NA
Missing	−14 218 (−28 224 to −212)	.047	−14 164 (−28 151 to −177)	.047
Marital status				
Not married	−4579 (−7310 to −1848)	.001	−4711 (−7491 to −1931)	.001
Married	0 [Reference]	NA	0 [Reference]	NA
Health insurance				
Currently have	14 572 (11 126-18 018)	<.001	14 577 (11 061-18 092)	<.001
Currently do not have	0 [Reference]		0 [Reference]	
Missing	3450 (−25 753 to 32 652)	.81	3755 (−26 334 to 33 843)	.80
Other chronic conditions, No.				
0	0 [Reference]	NA	0 [Reference]	NA
1	−6094 (−9384 to −2803)	<.001	−6214 (−9562 to −2866)	<.001
2	−18 169 (−21 826 to −14 511)	<.001	−18 449 (−22 054 to −14 844)	<.001
≥3	−22 734 (−31 733 to −13 736)	<.001	−23 548 (−32 415 to −14 680)	<.001
Missing	−10 837 (−38 967 to 17 294)	.45	−6888 (−36 542 to 22 766)	.65

Abbreviation: NA, not applicable.

^a^ Mean labor income losses associated with heart disease and stroke were estimated using the 2-part models and the postestimation *margin* commands. The *margin* commands were applied over all sociodemographic characteristics and other chronic conditions.

^b^ Includes non-Hispanic American Indian or Alaska Native persons, individuals with multiple races or other race, and unknown.

#### Labor Income Losses in Specific Groups of Age, Sex, and Race and Ethnicity

With increasing age, the MEs of heart disease on labor income became more pronounced, ranging from −$6364 (95% CI, −$10 630 to −$2098) for individuals aged 18 to 24 years to −$15 011 (95% CI, −$22 606 to −$7415) for individuals aged 45 to 54 years; likewise, the MEs of stroke also worsened from −$8789 (95% CI, −$14 193 to −$3385) for individuals aged 18 to 24 years to −$20 958 (95% CI, −$30 609 to −$11 308) for individuals aged 45 to 54 years. Despite the stronger MEs with age, the 95% CIs of marginal effects overlapped (Table 5). Similarly, although the MEs of heart disease and stroke on labor income were stronger for males than for females, MEs of −$16 288 (95% CI, −$24 849 to −$7726) for males with heart disease vs −$10 954 (95% CI, −$15 626 to −$6283) for females with heart disease and MEs of −$22 694 (95% CI, −$33 656 to −$11 731) for males with stroke vs −$15 182 (95% CI, −$21 274 to −$9090) for females with stroke, their 95% CIs overlapped. Such a pattern also existed when comparing the MEs of heart disease and stroke among Hispanic persons (heart disease: −$10 508 [95% CI, −$15 397 to −$5619]; stroke: −$14 630 [95% CI, −$21 019 to −$8241]), non-Hispanic Asian and Pacific Islander persons (heart disease: −$15 891 [95% CI, −$24 435 to −$7347] stroke: −$22 068 [95% CI, −$32 812 to −$11 324]), non-Hispanic Black persons (heart disease: −$11 614 [95% CI, −$16 996 to −$6231] stroke: −$16 108 [95% CI, −$23 141 to −$9076]), and non-Hispanic White persons (heart disease: −$14 274 [95% CI, −$21 191 to −$7357]; stroke: −$19 852 [95% CI, −$28 809 to −$10 895]). Overlapping 95% CIs of MEs at different age groups, sexes, or races and ethnicities may be partly explained by the small cell size issue: the number of persons with heart disease or stroke in some demographic groups was so small that the variance of estimated MEs was inevitably large.

**Table 5.** Labor Income Losses Associated With Heart Disease and Stroke at Specific Groups of Age Group, Sex, and Race and Ethnicity^a^

Characteristic	Model 1, heart disease (n = 12 110)		Model 2, stroke (n = 12 110)	
	Estimate (95% CI), $	*P* value	Estimate (95% CI), $	*P* value
Overall	−13 463 (−19 933 to −6993)	<.001	−18 716 (−27 077 to −10 356)	<.001
Age group, y				
18-24	−6364 (−10 630 to −2098)	.004	−8789 (−14 193 to −3385)	.002
25-34	−10 775 (−16 326 to −5223)	<.001	−15 032 (−22 350 to −7713)	<.001
35-44	−14 159 (−21 152 to −7166)	<.001	−19 791 (−28 834 to −10 749)	<.001
45-54	−15 011 (−22 606 to −7415)	<.001	−20 958 (−30 609 to −11 308)	<.001
55-64	−15 020 (−21 432 to −8607)	<.001	−20 722 (−29 065 to −12 379)	<.001
Sex				
Male	−16 288 (−24 849 to −7726)	<.001	−22 694 (−33 656 to −11 731)	<.001
Female	−10 954 (−15 626 to −6283)	<.001	−15 182 (−21 274 to −9090)	<.001
Race and ethnicity				
Hispanic	−10 508 (−15 397 to −5619)	<.001	−14 630 (−21 019 to −8241)	<.001
Non-Hispanic				
Asian/Pacific Islander	−15 891 (−24 435 to −7347)	<.001	−22 068 (−32 812 to −11 324)	<.001
Black	−11 614 (−16 996 to −6231)	<.001	−16 108 (−23 141 to −9076)	<.001
White	−14 274 (−21 191 to −7357)	<.001	−19 852 (−28 809 to −10 895)	<.001
Other^b^	−12 961 (−18 875 to −7047)	<.001	−17 972 (–25 572 to –10 373)	<.001

^a^ Labor income losses associated with heart disease and stroke at specific groups of age, sex, and race/ethnicity were estimated using the 2-part models and the post-estimation *margin* commands. The *margin* commands were applied at specific groups of age, sex, and race/ethnicity.

^b^ Includes non-Hispanic American Indian or Alaska Native persons, individuals with multiple races or other race, and unknown.

### Sensitivity Analysis

We compared results from models with and without interaction terms between heart disease or stroke and age group, sex, or race and ethnicity (eTable 3 in Supplement 1). AMEs of heart disease on labor income were very similar across models: −$13 147 (95% CI, −$19 652 to −$6642) for the model with interaction terms between heart disease and sex and −$13 247 (95% CI, −$20 601 to −$5894) for the model with interaction terms between heart disease and race and ethnicity compared with −$13 463 (95% CI, −$19 933 to −$6993) for the model without interaction terms. AMEs of stroke differed moderately across models: −$21 267 (95% CI, −$30 844 to −$11 689) for the model with interaction terms between stroke and sex and −$17 826 (95% CI, −$26 382 to −$9271) for the model with interaction terms stroke and race and ethnicity compared with −$18 716 (95% CI, −$27 077 to −$10 356) for the model without interaction terms. AMEs of heart disease and stroke were not estimable from models with interaction terms involving age group. Comparing results from models with and without diabetes (eTable 4 in Supplement 1), we found AMEs of heart disease and stroke were moderately higher in models without diabetes than in models with diabetes: −$14 718 (95% CI, −$21 133 to −$8303) vs −$13 463 (95% CI, −$19 933 to −$6993) for heart disease and −$19 336 (95% CI, −$27 752 to −$10 919) vs −$18 716 (95% CI, −$27 077 to −$10 356) for stroke.

### Total Labor Income Losses in the US

Given the prevalence of heart disease among persons aged 18 to 44 and 45 to 64 years in 2018 was 4.4% and 11.8%, respectively,^20^ we estimated the numbers of persons with heart disease aged 18 to 44 and 45 to 64 years in 2018 were 5.2 million and 9.9 million, respectively, by multiplying the prevalence of heart disease by corresponding Census population estimates.^21^ Since the mean labor income loss associated with heart disease was $13 463 and the number of persons aged 18 to 64 years with heart disease was 15.1 million, total labor income loss associated with heart disease in the US in 2018 would be approximately $203.3 billion. Similarly, we estimated total labor income loss associated with stroke would be approximately $63.6 billion.

## Discussion

This cross-sectional study found that annual labor income losses associated with heart disease were a mean of $13 463 and income losses associated with stroke were a mean of $18 716 in 2018 dollars, after adjustment for sociodemographic characteristics and other chronic conditions. Labor income losses associated with heart disease and stroke tended to worsen with age and were larger for males than for females and for non-Hispanic White persons and non-Hispanic Asian or Pacific Islander persons than for non-Hispanic Black persons and Hispanic persons, although their 95% CIs overlapped. Our estimates of labor income losses were not sensitive to model specifications: inclusion or exclusion of interaction terms between heart disease or stroke and sex or race and ethnicity and inclusion or exclusion of diabetes as a comorbidity.

To our knowledge, no prior studies have estimated labor income losses associated with heart disease and stroke using income data that capture both lower labor market participation and lower labor income among those with heart disease or stroke. A 2019 Canadian study^12^ found that the annual earnings losses associated with CVD events were $11 143 for cardiac arrest and $13 278 for stroke in 2012 Canadian dollars, equivalent to $9086 and $10 827 in 2018 US dollars,^22,23^ respectively. It is not surprising that these estimates are smaller than ours because they did not consider persons who had lower labor market participation because of heart disease and stroke conditions. Also, labor cost differences between the US and Canada and exchange rate fluctuations between 2012 and 2018 may partly explain the differences.

Our estimates contribute to calculating costs of lost productivity due to missed or lower labor market participation associated with CVD morbidity, a gap in the current literature.^2^ To put total labor income losses associated with morbidity of heart disease ($203.3 billion) and stroke ($63.6 billion) into perspective, we compare them with lost productivity costs of premature mortality from heart disease ($119.9 billion) and stroke ($19.4 billion).^2^ Since total labor income losses from morbidity of heart disease and stroke were substantial and far greater than lost productivity costs from mortality of heart disease and stroke, the current estimated total cost of CVD in 2018 ($378.0 billion),^2^ which only includes cost of productivity losses due to premature CVD mortality, substantially underestimates total cost of productivity losses, specifically associated with CVD morbidity. Labor income losses might also affect tax revenues: had labor income losses of $266.9 billion from morbidity of heart disease and stroke been averted, tax revenue might have increased $35.5 billion in 2018, given the mean tax rate of 13.3%.^24^ Both increased total cost of CVD and decreased tax revenue due to labor income losses associated with heart disease and stroke demonstrate the importance of CVD prevention in not only improving population health and controlling health care costs, but also supporting the financial security of US workers and the nation.

### Limitations

The study has several limitations. First, the study does not provide estimates of labor income losses by specific types of heart disease and stroke, although the specific types may differ in their impacts on patients’ health and labor market activities. Second, this study does not estimate labor income losses by onset and severity of heart disease and stroke, although the onset and severity of illness may be indirectly captured in terms of lower labor income among those with these conditions. Third, since both reference persons and their spouses or partners were included in the study sample, intrahousehold correlation may exist and result in underestimating SEs of the estimates. Fourth, heart disease and stroke and other chronic conditions in the PSID are self-reported not clinically verified, so this study may be subject to self-reporting bias. Fifth, the numbers of persons with heart disease and stroke in the PSID are small, preventing in-depth, subgroup analysis that may have shed more light on differences in labor income losses across subgroups. Sixth, this study treated other chronic conditions equally using an overall variable for number of other chronic conditions, possibly masking the differences in labor income losses associated with specific chronic conditions. Seventh, because this is a cross-sectional study, we cannot establish causation between heart disease or stroke and labor income losses. A longitudinal study that tracks heart disease and stroke incidence and labor income change could shed more light on causation.

## Conclusions

This cross-sectional study found that mean labor income losses associated with morbidity of heart disease and stroke among persons aged 18 to 64 years in the US in 2018 were $13 463 for heart disease and $18 716 for stroke. The estimated corresponding total labor income losses were $203.3 billion for heart disease and $63.6 billion for stroke, which are far greater than costs of productivity losses from premature mortality from these conditions. Comprehensive estimation of total costs of CVD may assist decision-makers in assessing benefits from averted premature mortality and morbidity from CVD and allocating resources to the prevention, management, and control of CVD.

## Notes

Supplement 1. **eTable 1.** Parameter Estimates for the 2-Part Model of Heart Disease **eTable 2.** Parameter Estimates for the 2-Part Model of Stroke **eTable 3.** Mean Labor Income Losses Associated with Heart Disease and Stroke Estimated from Models With and Without Interaction Terms **eTable 4.** Mean Labor Income Losses Associated with Heart Disease and Stroke Estimated from Models With and Without Diabetes as a Comorbidity

Supplement 2.
Data Sharing Statement
