# Supplementary material for: Labor Income Losses Associated With Heart Disease and Stroke From the 2019 Panel Study of Income Dynamics
Source: JAMA Netw Open. 2023 Mar 13;6(3):e232658. doi: 10.1001/jamanetworkopen.2023.2658 (PMC10011934; doi:10.1001/jamanetworkopen.2023.2658)
Supplement: Supplement 1. — eTable 1. Parameter Estimates for the 2-Part Model of Heart Disease eTable 2. Parameter Estimates for the 2-Part Model of Stroke eTable 3. Mean Labor Income Losses Associated with Heart Disease and Stroke Estimated from Models With and Without Interaction Terms eTable 4. Mean Labor Income Losses Associated with Heart Disease and Stroke Estimated from Models With and Without Diabetes as a Comorbidity [file jamanetwopen-e232658-s001.pdf]

Supplemental Online Content

Luo F, Chapel G, Ye Z, Jackson SL, Roy K. Labor income losses associated with heart disease and stroke from the 2019 Panel Study of Income Dynamics. *JAMA Netw Open*. 2023;6(3):e232658. doi:10.1001/jamanetworkopen.2023.2658

eTable 1. Parameter Estimates for the 2-Part Model of Heart Disease

eTable 2. Parameter Estimates for the 2-Part Model of Stroke

eTable 3. Mean Labor Income Losses Associated with Heart Disease and Stroke Estimated from Models With and Without Interaction Terms

eTable 4. Mean Labor Income Losses Associated with Heart Disease and Stroke Estimated from Models With and Without Diabetes as a Comorbidity

This supplemental material has been provided by the authors to give readers additional information about their work.

© 2023 Luo F et al. *JAMA Network Open.*

eTable 1. Parameter Estimates for the 2-Part Model of Heart Disease[a]

Part 1: Model the probability of having positive labor income
Probit Model

	Coefficient	Standard Error	*P*-value	95% Confidence Interval	
Heart Disease	-0.505	0.107	0	-0.719	-0.290
Age Group (Reference: 18-24 Years)					
25-34 Years	-0.392	0.110	0.001	-0.612	-0.172
35-44 Years	-0.520	0.122	0	-0.764	-0.276
45-54 Years	-0.510	0.116	0	-0.742	-0.278
55-64 Years	-0.946	0.111	0	-1.168	-0.724
Female (Reference: Male)	-0.433	0.041	0	-0.516	-0.351
Race and Ethnicity (Reference: Non-Hispanic White)					
Hispanic	-0.006	0.074	0.938	-0.154	0.143
Non-Hispanic Asian or Pacific Islander	0.032	0.131	0.809	-0.229	0.292
Non-Hispanic Black	-0.106	0.066	0.112	-0.236	0.025
Other	-0.177	0.087	0.046	-0.351	-0.003
Region (Reference: Northeast)					
North Central	0.024	0.076	0.756	-0.128	0.176
South	-0.076	0.082	0.361	-0.240	0.089
West	-0.129	0.082	0.122	-0.293	0.035
Outside US	-0.512	0.193	0.01	-0.897	-0.128
Education (Reference: At Least Some College Education)					
Below College	-0.418	0.052	0	-0.521	-0.314
Missing	0.119	0.215	0.582	-0.310	0.548
Not Married (Reference: Married)	0.061	0.045	0.176	-0.028	0.151
Health Insurance (Reference: Currently Do Not Have)					
Currently Have	0.088	0.085	0.305	-0.082	0.259
Missing	0.054	0.432	0.9	-0.808	0.917
Other Chronic Conditions, No. (Reference: 0)					
1	-0.237	0.041	0	-0.319	-0.155
2	-0.520	0.062	0	-0.644	-0.397
3+	-0.901	0.083	0	-1.066	-0.736
Missing	-0.450	0.422	0.289	-1.292	0.391
Constant	2.078	0.152	0	1.775	2.381

© 2023 Luo F et al. *JAMA Network Open.*

eTable 1. Parameter Estimates for the 2-Part Model of Heart Disease (Continued)

Part 2: Model labor income among those who reported positive labor income
Generalized Linear Model

	Coefficient	Standard Error	*P*-value	95% Confidence Interval	
Heart Disease	-0.153	0.082	0.065	-0.317	0.010
Age Group (Reference: 18-24 Years)					
25-34 Years	0.402	0.057	0.000	0.290	0.515
35-44 Years	0.640	0.061	0.000	0.518	0.763
45-54 Years	0.701	0.065	0.000	0.572	0.831
55-64 Years	0.622	0.067	0.000	0.488	0.755
Female (Reference: Male)	-0.487	0.019	0.000	-0.526	-0.449
Race and Ethnicity (Reference: Non-Hispanic White)					
Hispanic	-0.308	0.032	0.000	-0.372	-0.243
Non-Hispanic Asian or Pacific Islander	0.114	0.083	0.175	-0.052	0.281
Non-Hispanic Black	-0.227	0.033	0.000	-0.292	-0.162
Other	-0.130	0.033	0.000	-0.196	-0.064
Region (Reference: Northeast)					
North Central	-0.182	0.054	0.001	-0.290	-0.075
South	-0.132	0.038	0.001	-0.207	-0.057
West	-0.116	0.042	0.008	-0.201	-0.032
Outside US	-0.364	0.323	0.263	-1.008	0.280
Education (Reference: At Least Some College Education)					
Below College	-0.530	0.026	0.000	-0.583	-0.478
Missing	-0.299	0.150	0.050	-0.597	-0.001
Not Married (Reference: Married)	-0.113	0.027	0.000	-0.167	-0.059
Health Insurance (Reference: Currently Do Not Have)					
Currently Have	0.331	0.048	0.000	0.236	0.426
Missing	0.081	0.373	0.828	-0.662	0.825
Other Chronic Conditions, No. (Reference: 0)					
1	-0.063	0.028	0.030	-0.119	-0.006
2	-0.269	0.045	0.000	-0.359	-0.180
3+	-0.228	0.133	0.092	-0.493	0.038
Missing	-0.100	0.346	0.774	-0.790	0.591
Constant	10.740	0.086	0.000	10.568	10.912

[a] The number of observations used in the regressions for the two-part model is 12,110.

© 2023 Luo F et al. *JAMA Network Open.*

eTable 2. Parameter Estimates for the 2-Part Model of Stroke[a]

Part 1: Model the probability of having positive labor income
Probit Model

	Coefficient	Standard Error	*P*-value	95% Confidence Interval	
Stroke	-0.757	0.145	0.000	-1.046	-0.469
Age Group (Reference: 18-24 Years)					
25-34 Years	-0.389	0.111	0.001	-0.610	-0.168
35-44 Years	-0.526	0.122	0.000	-0.769	-0.283
45-54 Years	-0.514	0.117	0.000	-0.748	-0.281
55-64 Years	-0.951	0.110	0.000	-1.171	-0.731
Female (Reference: Male)	-0.418	0.041	0.000	-0.501	-0.336
Race and Ethnicity (Reference: Non-Hispanic White)					
Hispanic	-0.008	0.076	0.921	-0.160	0.145
Non-Hispanic Asian or Pacific Islander	0.043	0.127	0.735	-0.210	0.297
Non-Hispanic Black	-0.096	0.065	0.144	-0.225	0.034
Other	-0.160	0.088	0.075	-0.336	0.016
Region (Reference: Northeast)					
North Central	0.026	0.075	0.729	-0.124	0.176
South	-0.072	0.082	0.383	-0.234	0.091
West	-0.117	0.081	0.154	-0.280	0.045
Outside US	-0.503	0.197	0.013	-0.895	-0.111
Education (Reference: At Least Some College Education)					
Below College	-0.417	0.052	0.000	-0.521	-0.313
Missing	0.119	0.217	0.585	-0.313	0.551
Not Married (Reference: Married)	0.055	0.046	0.238	-0.037	0.146
Health Insurance (Reference: Currently Do Not Have)					
Currently Have	0.084	0.086	0.334	-0.088	0.256
Missing	0.061	0.437	0.889	-0.811	0.933
Other Chronic Conditions, No. (Reference: 0)					
1	-0.239	0.041	0.000	-0.320	-0.157
2	-0.534	0.059	0.000	-0.653	-0.416
3+	-0.933	0.082	0.000	-1.097	-0.768
Missing	-0.135	0.417	0.748	-0.966	0.697
Constant	2.068	0.154	0.000	1.760	2.376

© 2023 Luo F et al. *JAMA Network Open.*

eTable 2. Parameter Estimates for the 2-Part Model of Stroke (Continued)

Part 2: Model labor income among those who reported positive labor income
Generalized Linear Model

	Coefficient	Standard Error	*P*-value	95% Confidence Interval	
Stroke	-0.193	0.111	0.087	-0.414	0.029
Age Group (Reference: 18-24 Years)					
25-34 Years	0.402	0.057	0.000	0.289	0.515
35-44 Years	0.639	0.062	0.000	0.516	0.762
45-54 Years	0.699	0.065	0.000	0.570	0.829
55-64 Years	0.617	0.067	0.000	0.483	0.750
Female (Reference: Male)	-0.485	0.019	0.000	-0.523	-0.447
Race and Ethnicity (Reference: Non-Hispanic White)					
Hispanic	-0.307	0.032	0.000	-0.372	-0.242
Non-Hispanic Asian or Pacific Islander	0.115	0.084	0.174	-0.052	0.283
Non-Hispanic Black	-0.227	0.033	0.000	-0.293	-0.162
Other	-0.128	0.032	0.000	-0.191	-0.065
Region (Reference: Northeast)					
North Central	-0.182	0.054	0.001	-0.290	-0.074
South	-0.131	0.037	0.001	-0.206	-0.057
West	-0.116	0.042	0.007	-0.200	-0.032
Outside US	-0.363	0.324	0.266	-1.008	0.283
Education (Reference: At Least Some College Education)					
Below College	-0.531	0.026	0.000	-0.583	-0.478
Missing	-0.298	0.149	0.050	-0.595	0.000
Not Married (Reference: Married)	-0.114	0.027	0.000	-0.168	-0.060
Health Insurance (Reference: Currently Do Not Have)					
Currently Have	0.332	0.048	0.000	0.237	0.428
Missing	0.087	0.378	0.818	-0.667	0.842
Other Chronic Conditions, No. (Reference: 0)					
1	-0.065	0.029	0.028	-0.122	-0.007
2	-0.271	0.045	0.000	-0.360	-0.182
3+	-0.236	0.133	0.081	-0.502	0.030
Missing	-0.109	0.340	0.749	-0.788	0.569
Constant	10.738	0.087	0.000	10.565	10.911

[a] The number of observations used in the regressions for the two-part model is 12,110.

© 2023 Luo F et al. *JAMA Network Open.*

eTable 3. Mean Labor Income Losses Associated with Heart Disease and Stroke Estimated from Models With and Without Interaction Terms

	Heart Disease			Stroke	
	Estimate, $ (95% CI)	*P*-value		Estimate, $ (95% CI)	*P*-value
Model without Interaction Terms	-13,463 (-19,933 to -6,993)	<.001	Model without Interaction Terms	-18,716 (-27,077 to -10,356)	<.001
Model with Interaction Terms between Heart Disease and Age Group	Not Estimable		Model with Interaction Terms between Stroke and Age Group	Not Estimable	
Model with Interaction Terms between Heart Disease and Sex	-13,147 (-19,652 to -6,642)	<.001	Model with Interaction Terms between Stroke and Sex	-21,267 (-30,844 to -11,689)	<.001
Model with Interaction Terms between Heart Disease and Race/Ethnicity	-13,247 (-20,601 to -5,894)	.001	Model with Interaction Terms between Stroke and Race/Ethnicity	-17,826 (-26,382 to -9,271)	<.001

© 2023 Luo F et al. *JAMA Network Open.*

eTable 4. Mean Labor Income Losses Associated with Heart Disease and Stroke Estimated from Models With and Without Diabetes as a Comorbidity

	Heart Disease			Stroke	
	Estimate, $ (95% CI)	*P*-value		Estimate, $ [95% CI]	*P*-value
Model with Diabetes as a Comorbidity	-13,463 (-19,933 to -6,993)	<.001	Model with Diabetes as a Comorbidity	-18,716 (-27,077 to -10,356)	<.001
Model without Diabetes as a Comorbidity	-14,718 (-21,133 to -8,303)	<.001	Model without Diabetes as a Comorbidity	-19,336 (-27,752 to -10,919)	<.001

© 2023 Luo F et al. *JAMA Network Open.*
