# Supplementary material for: Labor Income Losses Associated With Heart Disease and Stroke From the 2019 Panel Study of Income Dynamics
Source: JAMA Netw Open. 2023 Mar 13;6(3):e232658. doi: 10.1001/jamanetworkopen.2023.2658 (PMC10011934; doi:10.1001/jamanetworkopen.2023.2658)
Supplement: Supplement 2. — Data Sharing Statement [file jamanetwopen-e232658-s002.pdf]

Data Sharing Statement

Luo. Labor Income Losses Associated With Heart Disease and Stroke From the 2019 Panel Study of Income Dynamics. *JAMA Netw Open*. Published March 13, 2023. doi:10.1001/jamanetworkopen.2023.2658

Data
Data available: Yes
Data types: Deidentified participant data, Data (not involving human participants), Data dictionary
How to access data: https://psidonline.isr.umich.edu/
When available: With publication

Supporting Documents
Document types: Statistical/analytic code
How to access documents: hto1@cdc.gov
When available: With publication

Additional Information
Who can access the data: readers who are interested in this study
Types of analyses: for any purpose
Mechanisms of data availability: after approval of a proposal
